# A randomized pilot study of a comprehensive postoperative exercise program compared with usual care following primary total hip arthroplasty in subjects less than 65 years of age: feasibility, selection of outcome measures and timing of assessment

**DOI:** 10.1186/1471-2474-15-192

**Published:** 2014-06-02

**Authors:** Lauren A Beaupre, Edward CO Masson, Barbara J Luckhurst, Orfan Arafah, Gregory J O’Connor

**Affiliations:** 1 Department of Physical Therapy, 2-50 Corbett Hall, University of Alberta, Edmonton, AB T6G 2G4, Canada; 2 Division of Orthopaedic Surgery, University of Alberta, 1F1.52 WMC, 8440-112 St, Edmonton, AB T6G 2B7, Canada; 3 Department of Rehabilitation, Royal Alexandra Hospital, Alberta Health Services, 1F1.52 WMC, 8440-112 St, Edmonton, AB T6G 2B7, Canada

**Keywords:** Total hip arthroplasty, Rehabilitation, Performance-based, Health related quality of life, Complex interventions

## Abstract

**Background:**

Total Hip Arthroplasty (THA) is being used more commonly in younger higher demand patients. The purpose of this randomized pilot study was to explore a) feasibility of comprehensive postoperative rehabilitation compared to usual care following primary THA in subjects <65 years, b) appropriate outcome measures including performance-based measures and c) timing of assessments.

**Methods:**

21 subjects who underwent primary THA were randomized to receive a three-month out-patient rehabilitation program (Intervention) or usual postoperative care (Control). Subjects were assessed preoperatively, six-weeks postoperatively (Pre-intervention) and four and 12 months postoperatively (Post-intervention). Self-report measures were the Western Ontario McMaster Osteoarthritis Index (WOMAC) and Rand 36-Item Health Survey (RAND-36). Performance-based measures included lower extremity strength, walking speed and endurance, and gait laboratory assessment.

**Results:**

Ten Control and 11 Intervention subjects with an average age of 53.4 (SD9.3) years were randomized. All Intervention subjects completed the program without adverse effects. Although no statistically significantly results were reported, four months postoperatively, Intervention subjects had clinically important differences (CID) in strength compared with Control subjects. Walking endurance, WOMAC and RAND scores improved significantly with no CID noted between groups. Ten (48%) subjects reported a ceiling effect on the WOMAC (9 (43%) subjects on Pain; 1 (5%) subject on Function). No group CID were noted in gait measures.

**Conclusions:**

Our recommendations would be that performance-based strength measures should be considered for the primary outcome in this younger cohort. Because of the ceiling effects with WOMAC Pain, a different pain measure is indicated. Other more challenging functional performance-based tests should be considered such as a more prolonged endurance test. There is merit in one-year follow-up as strength improved after four months in both groups.

## Background

Total Hip Arthroplasty (THA) significantly improves health-related quality of life (HRQL) for those with end-stage osteoarthritis (OA) [1], and is now used in younger patients (i.e., <65 years old) with debilitating joint disease. Due to higher activity levels [2], younger subjects may place higher demands on the THA.

Postoperative exercise programs are associated with increased strength and improved gait [3,4], but can be considered complex interventions [5]. When studies of these programs are undertaken, consideration must be given not only to the feasibility of the exercise program, but also to how best measure the impact of the rehabilitation intervention including selection of appropriate outcomes and outcome measures and timing of assessment [6]. Investigators should first consider smaller pilot studies before undertaking definitive randomized clinical trials (RCT) to maximize the likelihood of a successful clinical trial [5].

As an example, self-report measures, which are frequently used to assess recovery after THA, may not be adequately responsive in younger patients as ceiling effects (i.e., subjects achieve maximum scores) have been reported [7]. Performance-based measures may be more useful in assessing younger subjects [8]; however recent reports indicate that further assessment are needed to determine which performance-based measures are best suited for this population [9].

The objectives of this pilot were to explore 1) the feasibility of a comprehensive postoperative exercise intervention (Intervention) compared to usual postoperative care (Control) in subjects younger than 65 years following a primary THA 2) appropriate self-report and/or performance-based outcome measures, and 3) timing of assessments to evaluate the full program impact. Following this evaluation, we planned to undertake a definitive adequately powered RCT. We hypothesized that 1) the program would be well-tolerated, 2) performance-based measures would be more appropriate than patient-reported measures for this younger cohort and 3) that longer-term follow-up after program cessation would provide added value to the assessment.

## Methods

### Design

This was a single blind, randomized pilot study to determine feasibility (i.e., the ability of patients to undertake and complete the program as designed) and to assess appropriate outcomes and timing of assessment. Simple randomization was used to prevent bias in selecting which subjects underwent the planned intervention. Subjects provided signed informed consent and ethics approval was received from the University of Alberta Health Research Ethics Board (B-091199-RAH). Subjects were assigned to Intervention or Control groups using computer-generated randomization. Randomization codes were sealed in consecutively numbered opaque envelopes that were opened at hospital discharge.

### Sample size

As clinically important differences (CID) were known for the validated outcome measures that were selected for this appropriateness evaluation [7,10,11], 10 subjects per group were deemed adequate to detect CID if they occurred. We also wanted to determine if ceiling effects occurred in self-report measures in this younger cohort. Data were collected on the following performance-based measures: strength, endurance and gait measures to determine if performance measures were better suited to assess outcomes in this younger patient group. These pilot data could then inform power calculations for the RCT.

### Study population

Subjects were less than 65 years old, had recently undergone primary unilateral THA using a direct lateral (Hardinge) approach, which involves splitting the gluteus medius muscle during surgery. Subjects lived in the metropolitan area so that they could attend the program. Those subjects for whom the surgeon recorded a primary diagnosis of developmental dysplasia of the hip were excluded. Subjects were recruited at the Pre-Admission Clinic by a research associate who explained the study and obtained informed consent.

Surgeries were performed by experienced orthopaedic surgeons who routinely used the Hardinge approach. All participating surgeons (n = 4) performed greater than 50 THA per year and all surgeries occurred in a high volume (>1500 THA annually) tertiary center that followed a standardized inpatient clinical pathway.

### Intervention

All subjects received usual post-surgical care in the hospital and were discharged home with home exercises following a three to four day hospital stay. These home exercises involved ROM and isometric strengthening exercises for the hip musculature as well as encouraging walking with appropriate gait aids. Following the six week postoperative surgeon evaluation, Intervention subjects commenced the outpatient rehabilitation program. Sessions were approximately two and one-half hours in duration and included both aquatic and land-based components with a focus on strength and gait re-training (Appendix 1). Participants attended sessions two times/week for approximately three months and were encouraged to perform home exercises daily. Control subjects continued with usual care after their six-week appointment, which varied from the home exercises provided in hospital to community-based rehabilitation programs for a total of four to six sessions at patients’ discretion.

### Evaluation

Subjects were evaluated preoperatively, six weeks postoperatively (Pre-intervention), and at four and 12 months postoperatively (Post-intervention) by an evaluator blinded to group allocation. The evaluator assessed lower extremity (LE) strength using a hand held dynamometer as well as walking speed and endurance using the six minute walk test. The Western Ontario McMaster Osteoarthritis Index (WOMAC) and the RAND 36-item Health Survey (RAND-36) were used as self-report measures to evaluate health-related quality of life (HRQL). Gait kinematics were assessed in a gait laboratory six weeks postoperatively (Pre-intervention), and four and 12 months postoperatively (Post-intervention) by an evaluator blinded to group allocation. In addition, subjects were asked about adverse events related to both the medical care of their THA as well any adverse events associated with rehabilitation (e.g., falls, increased pain, inability to perform the program, missed program sessions).

#### Outcome measures

*Strength* of hip flexion, extension, abduction, internal and external rotation was measured in pounds of force utilizing a hand held dynamometer (microFET2™ Digital Handheld Muscle Tester; Hoggan Scientific, LLC) [11]. Abduction strength was not measured six-weeks postoperatively as subjects were not allowed to perform resisted abduction six weeks after a Hardinge surgical approach. These measures have been shown to be reliable with changes of greater than 10% representing a conservative estimate of true measurement differences [12]. Thus, we used differences of 10% as representing CID for strength measurements.

The *Six-Minute Walk test* assessed gait speed and endurance [10], with subjects able to use aids as required. The test was performed along indoor corridors that completed a full square, so that subjects could continue walking in the same direction. A rolling meter stick was held by the research associate who followed the subject along the course to measure the distance walked. The six-minute walk test has been used in this population previously and was chosen as it reflects both speed and walking endurance rather than focusing on a single aspect of gait [13]. The minimal CID is reported to be 61.34 meters [10].

*Gait Analysis:* An eight-camera passive-marker system, with three floor-mounted force plates was utilized. Subjects ambulated on a 10-meter walkway for three complete gait cycles. Velocity, cadence, stride length and width were recorded. Velocity measured the distance walked in meters/seconds while cadence measured the number of steps/minute. Stride length, measured in meters, was the distance between right foot heel strike to next right foot heel strike. Stride width was the distance between legs in the stance phase. All gait measurements were reported in percentages relative to normalized values, which were pre-determined in this gait laboratory using 14 adult subjects without LE dysfunction. Kinematic values greater than one indicated values greater than normal values while kinematic values less than one indicated values less than normal values.

The *WOMAC Osteoarthritis Index,* a reliable and valid disease-specific questionnaire for assessing THA outcomes was used to measure hip pain and function [7]. This measure has been extensively used in this population and is responsive to changes reported postoperatively. The CID for pain has been established at 21.38 points while the CID for function has been established at 11.9 points [7].

The *RAND-36*[14], is a 36-item generic health status questionnaire that has identical items and health dimensions as the SF-36, but does not have licensing fee. The RAND-36 was used to determine overall health status. The CID has been estimated for all eight dimensions of health and vary from 18.99 points for the dimension of Physical Function to 38.09 points for the dimension of Bodily Pain [7].

### Data analysis

Descriptive analyses were undertaken with independent T-tests or chi square tests while comparative analyses were performed using two-way repeated measures Analysis of Variance (ANOVA). Strength measures were reported as mean percentage change in strength between each measurement interval: a) preoperative to pre-intervention, b) pre-intervention to four months (post-intervention) and c) four months (post-intervention) to 12-months (post-intervention) with the exception of abduction. Abduction was reported as mean percentage change between a) preoperative and four months (post-intervention) and b) four months (post-intervention) to 12-months (post-intervention). All analyses were undertaken using Predictive Analytics SoftWare (PASW) version 18.0 (SPSS Inc., Chicago, Illinois, USA) with a level of significance set at p < 0.05.

## Results

### Demographics

Twenty-one subjects were randomized – 10 Control and 11 Intervention subjects. Participants’ average age was 53.4 (Standard Deviation [SD] 9.3) years; similar numbers of males and females participated, but the Control group was 70% male while the Intervention group was 64% female (Table 1). Of the 21 subjects, 13 (62%) had two or fewer co-morbid conditions with no group differences noted (p = 0.35). Fifteen (75%) subjects worked full-time preoperatively.

**Table 1:** Preoperative characteristics of 21 subjects randomized to a postoperative exercise intervention or usual care following primary total hip arthroplasty

	**Intervention**	**Control**	**P-value**
	**(n = 11)**	**(n = 10)**	
***Demographics***			
Mean age (SD)	51.7 (8.3)	55.9 (9.9)	0.30
Number of females n (%)	7 (64)	3 (30)	0.20
Working full time n (%)	7 (64)	8 (80)	0.11
***Performance-based measures***			
Strength in pounds of force			
Mean hip flexion (SD)	25.3 (8.5)	28.6 (7.0)	0.37
Mean hip extension (SD)	21.3 (7.2)	28.1 (13.7)	0.21
Mean hip external rotation (SD)	13.8 (5.0)	16.8 (5.5)	0.22
Mean hip internal rotation (SD)	13.1 (3.4)	16.3 (5.7)	0.16
Mean abduction (SD)	22.1 (8.3)	24.5 (6.7)	0.50
Mean six minute walk test in meters (SD)	402.2 (291.4)	472.2 (373.0)**	0.65
***Self- report measures***			
WOMAC*			
Mean pain score (SD)	46.0 (12.4)	55.6 (13.2)	0.13
Mean function score (SD)	51.0 (14.8)	55.1 (14.0)	0.56
RAND-36*			
Mean physical function score (SD)	27.8 (13.9)	40.0 (23.3)**	0.20
Mean physical role score (SD)	13.9 (25.3)	43.8 (37.2)**	0.07
Mean bodily pain score (SD)	26.5 (14.4)	34.9 (12.9)	0.22
Mean general health appendix score (SD)	78.3 (16.5)	79.0 (19.9)	0.94
Mean vitality score (SD)	48.5 (17.0)	53.8 (23.3)	0.59
Mean emotional role score (SD)	63.3 (42.9)	87.5 (24.8)**	0.09
Mean social function score (SD)	53.8 (32.3)	75.0 (14.9)**	0.16
Mean mental health score (SD)	76.4 (15.8)	83.0 (11.7)	0.34

Legend: SD = Standard Deviation; WOMAC = Western Ontario McMaster Osteoarthritis Index; RAND-36 = RAND 36-Item Health Survey.

*Scores are rated as 0 = no health limitation – 100 = maximal health limitation.

**Exceeds clinically important difference.

### Baseline evaluation

Intervention subjects walked a shorter distance on the six-minute walk test, but had similar strength as Control subjects (Table 1). Although walking test differences surpassed the CID of 62 meters, they were not statistically significant in this small sample. Groups were also similar in patient-reported outcomes preoperatively except for three RAND-36 dimensions. Intervention subjects reported CID in Role Physical, Role Emotional and Social Function scores, suggesting that they were experiencing more health limitations than Control subjects (Table 1); however as expected with this small pilot study, these differences were not statistically significant.

### Program tolerance

All Intervention subjects were able to tolerate the intervention and all 11 subjects completed the three-month program without experiencing any adverse events.

### Postoperative performance-based measures

i) ***Strength*** Six weeks postoperatively, subjects had lower strength scores than preoperatively, with most changes representing true losses of strength based on apriori established CID (Table 2). At four months postoperatively, while both groups had CID improvements in strength, the Intervention subjects had higher mean changes than the Control group changes, all of which were much greater than the apriori CID of 10% between groups. At 12 months, only abduction and internal rotation had improved beyond CID in the intervention groups with the remaining values unchanged from four months. In contrast, Control subjects reported clinically important gains in strength in external rotation, abduction and extension between four and 12 months (Table 2). As expected, none of these differences were significant between groups in this small sample.

ii) ***Six Minute Walk Test*** Six weeks postoperatively, all subjects walked shorter distances than preoperatively (Table 3). Four months postoperatively, the Control group returned to walking preoperative distances, while the Intervention group surpassed preoperative measurements. Twelve-months postoperatively, both groups were walking similar distances and surpassed preoperative distances (Table 2). At no time did group difference surpass the CID of 62 meters, but all patients improved their walking distance well beyond the CID within 12 months of surgery.

iii) ***Gait*** At no evaluation were group differences noted in velocity, cadence, stride length or width (Table 3). All subjects improved in gait velocity, cadence and stride length between six weeks and four months with no further improvements at 12 months, but remained lower than the reference values. Stride width did not change and was greater than normal values for both groups (Table 3).

**Table 2:** Mean percentage change in strength of 21 subjects randomized to a postoperative exercise intervention or usual care following primary total hip arthroplasty

	**Mean% change from preop to 6 weeks (Pre-intervention)**		**Mean% change from 6 weeks (Pre-intervention) to 4 months (Post-intervention)**		**Mean% change from 4-months to 12-months postoperative**		
					**Post-intervention**		
	**Intervention**	**Control**	**Intervention**	**Control**	**Intervention**	**Control**	P-value for group effect*
	**(n = 11)**	**(n = 10)**	**(n = 11)**	**(n = 10)**	**(n = 11)**	**(n = 10)**	
**Performance-based measures**							
Hip flexion (SD)	−24.2 (31.7)	−7.7 (40.0)	73.8 (50.1)	39.8 (64.1)	−2.5 (11.8)	5.3 (19.1)	0.69
Hip extension (SD)	−13.2.9 (27.7)	−7.5 (52.3)	50.5 (26.1)	30.5 (67.3)	3.1 (30.2)	12.2 (26.8)	0.78
Hip External rotation (SD)	−52.2 (14.3)	−34.7 (36.4)	54.2 (33.2)	9.4 (21.6)	3.9 (26.3)	34.0 (24.2)	0.89
Hip internal rotation (SD)	−24.6 (46.9)	−33.6 (34.1)	46.7 (83.2)	35.7 (28.0)	14.2 (48.0)	−0.1 (22.4)	0.14
Abduction (SD)	-	-	37.8 (62.9)	6.2 (48.1)	13.0 (20.1)	16.5 (48.1)	0.25

Legend: SD = Standard Deviation.

*Analyzed using a 2-way Repeated Measures ANOVA.

**Table 3:** Performance-based and self- report measures of 21 subjects randomized to a postoperative exercise intervention or usual care following primary total hip arthroplasty 6 weeks postoperatively (Pre-intervention) and 4 and 12 months postoperatively (Post-intervention)

	**6-weeks postoperative**		**4-months postoperative**		**12-months postoperative**		
	**Pre-intervention**		**Post-intervention**		**Post-intervention**		
	**Intervention**	**Control**	**Intervention**	**Control**	**Intervention**	**Control**	P-value for group effect*
	**(n = 11)**	**(n = 10)**	**(n = 11)**	**(n = 10)**	**(n = 11)**	**(n = 10)**	
**Performance-based measures**							
Mean 6-minute walk test in meters (SD)	331.0 (6.4)	287.1 (84.8)	450.6 (61.4)	435.8 (99.2)	500.6 (99.6)	495.6 (75.6)	0.92
Gait measures**							
Mean velocity (SD)	0.55 (0.17)	0.46 (0.13)	0.72 (0.09)	0.77 (0.19)	0.75 (0.11)	0.84 (0.14)	0.96
Mean cadence (SD)	0.73 (0.15)	0.70 (0.10)	0.86 (0.08)	0.93 (0.11)	0.88 (0.08)	0.93 (0.08)	0.91
Mean stride length (SD)	0.74 (0.10)	0.64 (0.13)	0.83 (0.07)	0.83 (0.15)	0.85(0.08)	0.90 (0.11)	0.80
Mean stride width (SD)	1.2 (0.63)	1.3 (0.29)	1.1 (0.44)	1.1 (0.45)	1.2 (0.38)	1.2 (0.22)	0.90
**Self-report measures**							
WOMAC†							
Mean pain score (SD)	77.3 (17.8)	80.6 (15.7)	86.4 (14.2)	82.2 (10.0)	91.7 (13.2)	87.0 (18.9)	0.65
Mean function score (SD)	71.8 (12.8)	70.4 (17.6)	82.4 (13.5)	81.2 (1.7)	90.8 (12.7)	85.6 (15.6)	0.98
RAND-36†							
Mean physical function Score (SD)	42.7 (19.8)	33.5 (18.7)	67.1 (17.1)	64.4 (18.6)	82.8 (10.3)	73.3 (19.6)	0.48
Mean physical role score (SD)	15.9 (35.8)	13.9 (33.3)	63.6 (45.2)	66.7 (50.0)	22.9 (25.3)	75.0 (40.8)	0.78
Mean bodily pain score (SD)	49.5 (9.5)	41.0 (27.0)	71.5 (24.3)	56.8 (25.1)	82.3 (21.4)	73.4 (27.7)	0.31
Mean general health score (SD)	73.9 (21.7)	69.9 (23.1)	75.5 (5.9)	78.8 (17.2)	83.4 (12.1)	69.9 (16.7)	0.70
Mean vitality score (SD)	48.6 (15.2)	53.3 (18.9)	68.6 (19.5)	63.9 (19.3)	75.9 (15.5)	69.5 (24.3)	0.95
Mean emotional role score (SD)	36.4 (45.8)	59.3 (49.4)	93.9 (20.1)	77.8 (37.3)	88.9 (33.3)	80.0 (35.8)	0.63
Mean social function score (SD)	44.3 (25.2)	56.9 (36.5)	77.3 (23.6)	69.4 (27.3)	91.7 (16.5)	76.3 (24.6)	1.00
Mean mental health score (SD)	70.5 (15.2)	80.4 (19.8)	86.5 (10.2)	84.9 (14.5)	84.0 (10.4)	80.7 (20.1)	0.38

Legend: SD = Standard Deviation; WOMAC = Western Ontario McMaster Osteoarthritis Index; RAND-36 = RAND 36-Item Health Survey.

*Analyzed using a 2-way Repeated Measures ANOVA.

**Gait measures are listed as percentages relative to normal values. Values of less than 1 indicate that the study cohort was less than reference values; values greater than1 indicate that the study cohort was greater than normal values.

†Scores are listed as 0 = no health limitations - 100 = maximal health limitations.

### Patient-reported measures

i) Within six-weeks, WOMAC pain and function had improved significantly with no CID noted between groups (Table 3). Subjects reported further improvements within 12 months. Nine (43%) subjects (5 Intervention; 4 Control) reported a maximum improvement in pain by 12 months while 1 (5%) Intervention subject reported maximum improvement in function by 12 months.

ii) Subjects reported significant improvements on RAND-36 scores with the Intervention group reporting clinically importantly better scores in Social Function, General Health and Role Physical than the Control group at 12 months postoperatively (Table 3).

## Discussion

Our pilot study demonstrated that a comprehensive outpatient rehabilitation program is well-tolerated by patients less than 65 years of age who undergo THA. All subjects completed the intervention and no adverse reports were reported. Our findings also emphasize the need to carefully evaluate and select appropriate outcome measures. Although our pilot sample was small and did not achieve statistical significance, we measured CID between groups in strength measures only. Outcomes that have proven useful in other studies of THA did not detect any CID between our study groups, which were a young cohort of patients undergoing THA.

Using a hand held dynamometer, we were able to measure CID in all strength measures between groups at the initial post-intervention assessments [12]. Although both groups made substantial gains in strength over time, the mean changes measured in strength were much greater in the Intervention group, particularly at the four month evaluation.

Interestingly, this strength difference did not impact performance on the six-minute walk test, which has been shown to be a useful measure to assess outcomes following TJA [10]. No CID was measured between groups over time. The six minute walk test was selected because it measures both speed and endurance. However, in this younger group, more challenging performance-based functional tests may be required [4]. Use of a formal gait laboratory assessment also did not detect any CID between groups. This was a particularly disappointing finding as formal gait re-training was a component of the intensive rehabilitation program.

Similar to others [3,4], marked improvement occurred in self-reported HRQL over the first year following THA in our pilot study. Ceiling effects were reported in more than 40% of WOMAC Pain scores, but only 5% of WOMAC Function scores. However, with all subjects reporting substantial improvements in pain and function, the WOMAC was unable to discriminate between groups with group differences being well under the established CID [7].

Our study followed the recommendations of the Medical Research Council’s Guidelines on developing complex interventions [5]. Rehabilitation falls into the category of complex interventions because rehabilitation programs typically involve multiple components and may be delivered and evaluated in different formats [6,15,16]. Rehabilitation practitioners often do not adequately describe their intervention content or do not consider the impact of selected outcome measures and timing of the evaluation. Our goal with this pilot study was to determine the feasibility of our comprehensive program as well as to explore appropriate outcomes and outcome measures, and the timing of the evaluations prior to undertaking a definitive RCT.

Evaluation of the impact of the intervention both immediately after the intervention and again eight months later demonstrated that Intervention subjects maintained their earlier strength gains, but also demonstrated that Control subjects regained their strength more slowly over the first post-operative year in most hip musculature. Further evaluation is needed to determine if there is any long-term impact to the intensive rehabilitation program or if it allows earlier return to activity and work in this younger cohort.

This pilot study was a useful preliminary step in determining the feasibility of the intervention as well as determining appropriate outcome measures and assessment time points. Using a randomized design with blinded assessment of outcomes at multiple time points increases the likelihood that our findings were measuring the impact of the intervention. Although we found that the program was feasible and well-tolerated by our subjects, we also determined that commonly used self-report and performance-based outcome measures for subjects undergoing THA do not appear to be appropriate to detect differences in postoperative rehabilitation in this younger cohort. Thus, this initial pilot study circumvented a large and expensive randomized trial being undertaken with inappropriate outcome measures.

However, the sample was small and there were some baseline imbalances between groups in HRQL that might have affected our results. Although the Intervention group reported more overall preoperative health limitations, they surpassed the Control group in these same health dimensions at the one-year assessment. We were able to detect CID in strength measures, but cannot be assured that these findings will be re-produced in a large well-powered RCT. Further, we did not record the specific care provided to the Control group. In the future RCT, ‘usual care’ should be as standardized as possible and recorded to fully measure the differences between the usual care and intervention programs.

## Conclusions

Our recommendations following this pilot evaluation would be to consider performance-based strength measures as the primary outcomes for evaluating rehabilitation interventions of this nature. Based on the high proportion of ceiling effects with WOMAC Pain score, a different pain measure is indicated. Other more challenging functional performance-based tests or self-report measures that look at higher levels of activity should be considered to discriminate between groups. Perhaps a more prolonged endurance test would prove more useful in this younger cohort. Finally, there is merit in continuing to assess subjects out to one-year postoperatively as there were improvements in strength that continued after the program ended in both groups. Additional outcomes to consider based on our findings may be time to return to work and/or activity or number of postoperative falls based on the CID that were noted in strength at the initial post-intervention assessment. We are currently planning a large scale randomized study to assess the impact of the exercise program.

## Appendix 1: Young total hip rehabilitation program

The program had both an aquatic and land-based exercise components, which were held twice weekly with a maximum number of 10 subjects per class.

•Subjects performed the aquatic component first followed by the land exercises.

•The aquatic component was one hour in duration.

•Land-based component was 1.5 hours in duration.

Subjects commenced the program after their 6-week appointment and then continued the program until they were approximately 4 months post-operative. Subjects were instructed to use their cane for walking outside of the home until at least 3 months post-operative.

### Aquatic component’

The therapeutic pool was heated to 93 degrees Fahrenheit. The shallow end had one long step across the pool at each of 3 different depths – 2 foot, 3 foot and 4 foot. The 4 foot depth had a set of imbedded parallel bars and a continuous horizontal grab bar rail attached to the side-walls of the pool all the way around. At mid-point of the pool length the 4-foot depth sloped to the deep end.

Aquatic classes used all 3 different levels of steps and the entire length of the pool. As subjects improved they moved into shallower water. Activities included:

•walking forward, backwards, and side-ways

•stair-stepping forwards, backwards and sideways

•single leg heel raises and squats

•single leg balancing while moving the other leg and upper extremities

•side squats on stairs with opposite leg reaching to floor

•flutter kicking and alternate hip-knee flexion in prone while holding onto either side-wall bar or over parallel bars

•deep water running with pool noodles or aqua-belts for upright buoyancy

•ball tossing while single leg standing in a circle

### Land-based component

Prior to their land exercises, subjects did a 5 – 10 minute warm-up on stationery bicycles with zero-to-minimum resistance and the seat heights adjusted to ensure they did not flex their hips more than 90 degrees.

Initial activities included:

•heel slides for hip and knee flexion

•slider board abduction

•bridging

•core strengthening

•modified Thomas test for hip flexor stretching

•prone knee flexion

•sitting hamstring stretching

•quadriceps strengthening.

Activities were progressed to

•unassisted ambulation as able dependent on their hip abductor strength and ability to walk without limping

•closed kinetic chain exercises

◦ single leg balancing while moving upper extremities

◦ double and single leg wall squats with or without a ball at the back

◦ free-standing double and single leg heel raises and squats

◦ single leg wall pushes – i.e., standing on the operative leg while pushing the unaffected flexed hip and knee sideways against the wall to encourage weight transference onto the operative leg.

•balance and core strength while sitting on a therapy ball

•specific hip extensor, abductor and rotator muscle strengthening exercises such as: 

◦ bridging and then moving into hip abduction and adduction

◦ clam-shell for piriformis

◦ reverse clam-shell for posterior gluteus medius

◦ side-lying abduction for anterior gluteus medius and tensor fascia lata.

Resistance was added as tolerated (according to pain &/or resistance) with either elastic thera-bands or small sandbags.

All patients were video-taped while walking at assessment, re-assessments and discharge. The therapist used the anterior, posterior, and lateral views for gait-re-education purposes and as part of the clinical progress record. Soft-tissue manipulation, trigger point therapy, and positional releases were modified as required for those individual patients who continued to have soft tissue pain.

## Abbreviations

ANOVA: Analysis of variance; CID: Clinically important differences; LE: Lower extremity; OA: Osteoarthritis; RAND-36 – RAND: 36 item health survey; RCT: Randomized clinical trial; SD: Standard deviation; THA: Total hip arthroplasty; WOMAC: Western Ontario McMaster osteoarthritis index.

## Competing interests

The authors have no competing interests to declare.

## Authors’ contributions

LAB led study design, coordinated data collection and analysis and was the primary writer of the manuscript. ECOM assisted with study design and data collection and critically reviewed the manuscript. BJL assisted with study design and data collection and critically reviewed the manuscript. OA assisted with data collection and analysis and critically reviewed the manuscript. GJOC assisted with study design, attaining study funding, data collection and critically reviewed the manuscript. All authors read and approved the final manuscript.

## Authors’ information

Dr. Beaupre receives salary support from Alberta Innovates- Health Solutions as a Population Health Investigator and from the Canadian Institutes of Health Research as a New Investigator (Patient Oriented Research).

## Pre-publication history

The pre-publication history for this paper can be accessed here:

http://www.biomedcentral.com/1471-2474/15/192/prepub
